# Identification of *C*-mannosylation in a receptor tyrosine kinase AXL

**DOI:** 10.1093/glycob/cwae096

**Published:** 2024-12-03

**Authors:** Kento Mori, Takehiro Suzuki, Urara Waki, Soichiro Hayashi, Shigehito Kadono, Ryota Kawahara, Minae Takeuchi, Hayato Mizuta, Naoshi Dohmae, Ryohei Katayama, Siro Simizu

**Affiliations:** Department of Applied Chemistry, Faculty of Science and Technology, Keio University, Yokohama, Kanagawa 223-8522, Japan; Biomolecular Characterization Unit, RIKEN Center for Sustainable Resource Science, Wako, Saitama 351-0198, Japan; Department of Applied Chemistry, Faculty of Science and Technology, Keio University, Yokohama, Kanagawa 223-8522, Japan; Department of Applied Chemistry, Faculty of Science and Technology, Keio University, Yokohama, Kanagawa 223-8522, Japan; Department of Applied Chemistry, Faculty of Science and Technology, Keio University, Yokohama, Kanagawa 223-8522, Japan; Department of Applied Chemistry, Faculty of Science and Technology, Keio University, Yokohama, Kanagawa 223-8522, Japan; Department of Applied Chemistry, Faculty of Science and Technology, Keio University, Yokohama, Kanagawa 223-8522, Japan; Department of Applied Chemistry, Faculty of Science and Technology, Keio University, Yokohama, Kanagawa 223-8522, Japan; Division of Experimental Chemotherapy, Cancer Chemotherapy Center, Japanese Foundation for Cancer Research, Tokyo 135-8550, Japan; Biomolecular Characterization Unit, RIKEN Center for Sustainable Resource Science, Wako, Saitama 351-0198, Japan; Division of Experimental Chemotherapy, Cancer Chemotherapy Center, Japanese Foundation for Cancer Research, Tokyo 135-8550, Japan; Department of Applied Chemistry, Faculty of Science and Technology, Keio University, Yokohama, Kanagawa 223-8522, Japan

**Keywords:** AXL, Breast cancer, *C*-mannosylation, Receptor tyrosine kinase, Vasculogenic mimicry

## Abstract

*C*-mannosylation is a unique type of glycosylation in which a mannose is added to tryptophan in a protein. However, the biological function of *C*-mannosylation is still largely unknown. AXL is a receptor tyrosine kinase, and its overexpression contributes to tumor malignancy. The role of AXL in cancer cells is broad, including invasion, drug resistance, and vasculogenic mimicry formation. Although Trp320 of AXL was predicted to be *C*-mannosylated, it has not been confirmed. Here, we demonstrated that Trp320 of AXL is *C*-mannosylated, measured by mass spectrometry of recombinant AXL purified from various cancer cells. Furthermore, re-expression of *C*-mannosylation-deficient AXL in human breast cancer MDA-MB-231 cells lacking AXL by the CRISPR/Cas9 system resulted in reduction of vasculogenic mimicry formation. Interestingly, phosphorylation levels of AKT in *C*-mannosylation-deficient AXL re-expressing cells were comparable to those of parental and wild-type AXL re-expressing cells. These results represent the first discovery of *C*-mannosylation in a receptor tyrosine kinase and the possibility that *C*-mannosylation may affect AXL function, distinct from its downstream signaling in cancer cells.

## Introduction

*C*-mannosylation is one of the glycosylations in which a mannose attaches to the indole C2 carbon of the N-terminal tryptophan residue of the consensus amino acid sequence Trp-Xaa-Xaa-Trp/Cys (Xaa: any amino acid), via a C-C bond (Hofsteenge et al. 1994; de Beer et al. 1995; Krieg et al. 1998). *C*-mannosylation was first identified in human RNase2 from human urine in 1994 (Hofsteenge et al. 1994). The number of *C*-mannosylated proteins is about 40, and most of them are found in the thrombospondin type 1 repeat (TSR) superfamily or type 1 cytokine receptors (Niwa and Simizu 2018; Otani et al. 2018; Mizuta et al. 2019; Miura et al. 2021; Yoshimoto et al. 2021). *C*-mannosylation regulates protein functions, such as secretion, intracellular localization, and stability (Okamoto et al. 2017; Frank et al. 2020; Mizuta et al. 2023; Yoshimoto et al. 2023). There is only one example of a relationship between *C*-mannosylation and diseases. ADAMTSL1, one of the TSR superfamily members, is *C*-mannosylated at Trp^42^, and Trp42Arg mutation of ADAMTSL1 causes developmental glaucoma (Wang et al. 2009; Hendee et al. 2017). Dpy-19 is the *C*-mannosyltransferase for the TSR domain in *C. elegans* (Buettner et al. 2013). In mammals, there are four homologs of DPY19 (DPY19L1-L4), and DPY19L1 and DPY19L3 have *C*-mannosyltransferase activity (Niwa et al. 2016; Shcherbakova et al. 2017). However, while the functions and glycosyltransferases are reported for *C*-mannosylated proteins mainly in the TSR superfamily or type 1 cytokine receptors, the functions of *C*-mannosylation in other types of proteins remain unclear.

**Fig. 1:**
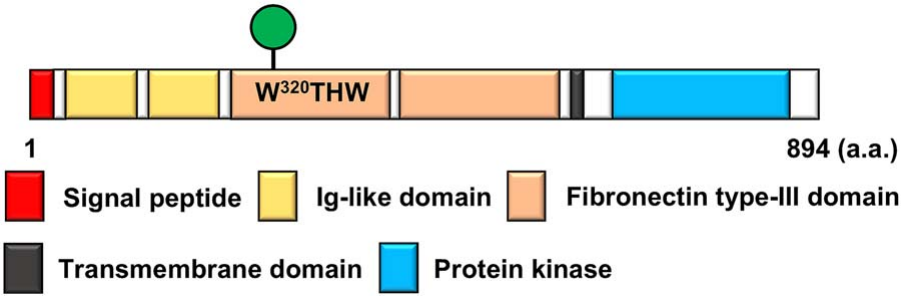
Schematic of human AXL. The Trp to be *C*-mannosylated is in the fibronectin type-III domain.

AXL is one of the receptor tyrosine kinases (RTKs) and belongs to the TAM receptor family (O'Bryan et al. 1991) (Fig. 1). AXL is activated by its ligand, GAS6, or a ligand-independent mechanism, and AXL activation induces the activation of several signaling pathway, including MAPK/ERK and PI3K/AKT (Feneyrolles et al. 2014). High expression of AXL in tumors is reported to be associated with a poor prognosis in patients (Feneyrolles et al. 2014). In human breast cancer MDA-MB-231 cells, inhibition of the interaction between AXL and GAS6 with monoclonal antibodies suppresses AXL activation and cell migration (Chang et al. 2021). Most non-small cell lung cancers (NSCLCs) with activating mutations in epidermal growth factor receptor (EGFR) respond to EGFR-tyrosine kinase inhibitors (EGFR-TKIs). Osimertinib is a 3rd-generation EGFR-TKI approved for the treatment of EGFR-mutated NSCLC and is also effective against T790M mutation-positive cases, frequently observed after treatment with 1^st^- or 2^nd^-generation EGFR inhibitors, such as gefitinib, erlotinib, or afatinib. However, some NSCLC patients show resistance to osimertinib treatment, and the previous study showed that one of the mechanisms of the resistance was the activation of the bypass growth signaling pathway by AXL (Taniguchi et al. 2019).

Vasculogenic mimicry (VM) is the formation of blood vessel-like structures with only cancer cells, and it is an alternative blood supply system for tumors, instead of angiogenesis (Folberg et al. 2000). VM has been observed in several cancers, such as melanoma, lung, ovarian, prostate, and breast cancer, and is associated with a poor clinical outcome (Maniotis et al. 1999; Shirakawa et al. 2001; Sood et al. 2001; Sharma et al. 2002). Therefore, VM is now expected to become a therapeutic target of cancer. In MDA-MB-231 cells, inhibition of AXL expression suppresses VM formation, cell invasion, and migration (Lim et al. 2020). However, the detailed mechanism of VM, especially the relationship to glycosylation, remains unknown. Moreover, Trp^320^ in the fibronectin type-III domain of AXL fits the consensus sequence of *C*-mannosylation (Trp^320^-Thr-His-Trp), suggesting the presence of it, but this has never been investigated.

Here, we first demonstrated *C*-mannosylation of AXL at Trp^320^ in various cancer cell lines. Furthermore, we showed that the *C*-mannosylation affects VM formation of MDA-MB-231 cells. Our study demonstrates the novel function of *C*-mannosylation through the effect of AXL functions and provides the possibility that *C*-mannosylation of AXL becomes the therapeutic target of cancers.

## Results

### AXL is *C*-mannosylated at Trp ^320^ in various cancer cell lines

**Fig. 2:**
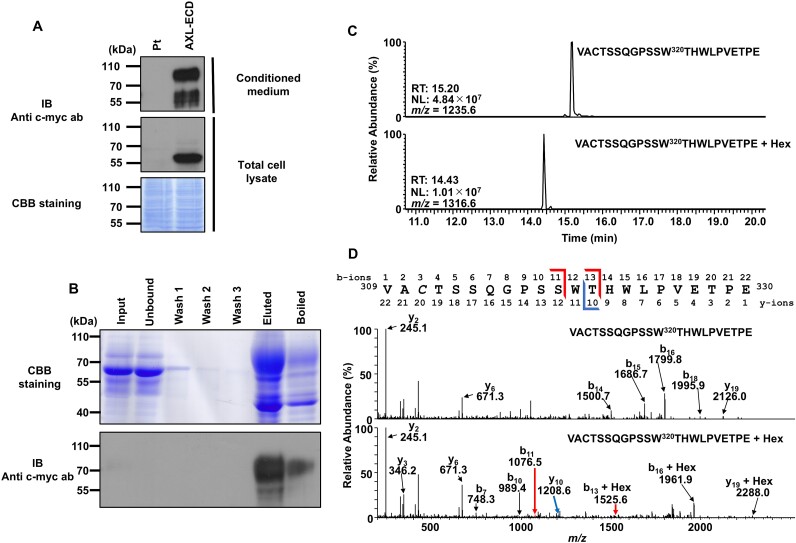
AXL is *C*-mannosylated at Trp^320^. (A) Establishment of AXL-ECD-overexpressing MDA-MB-231 cells. Parental (Pt) and AXL-ECD-overexpressing MDA-MB-231 cells were cultured in serum-free DMEM for 24 h. the cell lysates and conditioned media were electrophoresed and immunoblotted with anti-c-myc antibody. (B) Purification of recombinant AXL-ECD from AXL-ECD-overexpressing MDA-MB-231 cells. Cells were cultured in serum-free DMEM for 24 h, and conditioned medium was collected. The conditioned medium was concentrated by ultrafiltration and purified with Ni-NTA agarose. The samples were electrophoresed, and the bands were visualized by CBB staining. (C) Detection of hexosylated peptides including Trp^320^ of AXL. Purified recombinant AXL-ECD protein from MDA-MB-231 cells was digested by trypsin and endoproteinase Glu-C, and the resulting peptides were analyzed by LC–MS/MS. The unmodified and hexosylated ^309^VACTSSQGPSSWTHWLPVETPE^330^ peptides were detected as *m*/*z* = 1235.6 and 1316.6, respectively. (D) The MS/MS spectra of unmodified and hexosylated ^309^VACTSSQGPSSWTHWLPVETPE^330^ peptides. The observed peaks of these fragments were the indicated b- and y-ions. Italicized *C* indicates the propionamidated cysteine.

To identify the *C*-mannosylation of AXL, we established an MDA-MB-231 cell line that overexpressed the extracellular domain of AXL (AXL-ECD) (Fig. 2A). Recombinant AXL-ECD was purified from the conditioned media (Fig. 2B). Purified AXL-ECD was digested with trypsin and endoproteinase Glu-C, followed by LC–MS/MS analysis. We detected 2 peptides with *m/z* values of 1235.6 and 1316.6 (*z* = 2), which corresponded to unmodified and mono-hexosylated forms of ^309^VACTSSQGPSSWTHWLPVETPE^330^ peptide, respectively (Fig. 2C). To clarify the hexosylation site of AXL, MS/MS analysis of ^309^VACTSSQGPSSWTHWLPVETPE^330^ peptides was performed. The MS/MS spectrum of the modified peptide showed that the attachment of hexose was observed in the b13 but not b11 and y10 ions (Fig. 2D). These results suggested that AXL is *C*-mannosylated at Trp^320^.

**Fig. 3:**
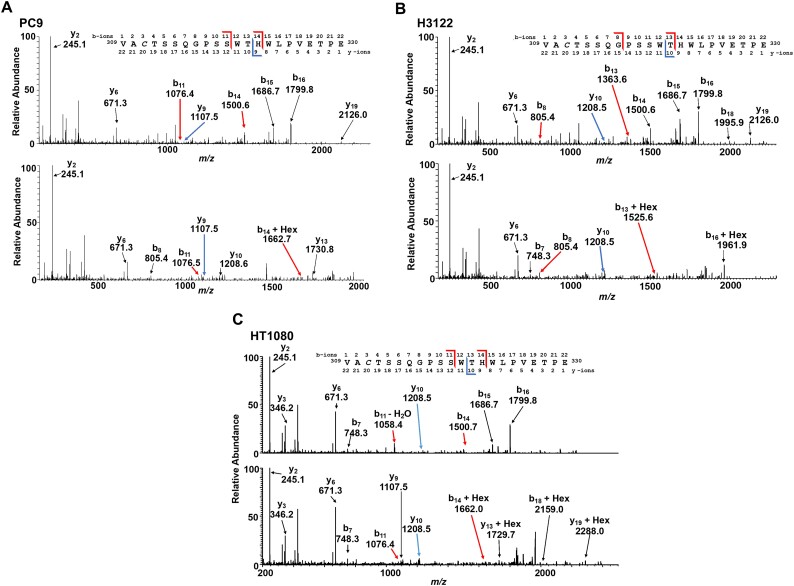
AXL is *C*-mannosylated in various cancer cell lines. (A-C) the MS/MS spectra of unmodified and hexosylated ^309^VACTSSQGPSSWTHWLPVETPE^330^ peptides from AXL-ECD-overexpressing PC9 (A), H3122 (B), and HT1080 (C) cells. The observed peaks of these fragments were the indicated b- and y-ions. Italicized *C* indicates the propionamidated cysteine.

To detect the *C*-mannosylation of AXL in other cell lines, we established AXL-ECD-overexpressing cells, purified recombinant AXL-ECD, and performed LC–MS/MS analysis in 3 cancer cell lines: human NSCLC PC9 and H3122 and human fibrosarcoma HT1080 (Fig. 3A-C, Fig. S1). In H3122 and HT1080 cell lines, *C*-mannosylation of Trp^320^ in AXL was detected (Fig. 3B and C). In PC9 cells, LC–MS/MS result demonstrated that Trp^320^ or Thr^321^ was hexosylated (Fig. 3A). If this hexosylation was an *O*-glycosylation at Thr^321^, it would be inconsistent with the detection of the modified peptide fragment in the MS/MS analysis. This suggested that this glycosylation in PC9 cells occurred at Trp^320^. These results indicated that AXL is *C*-mannosylated at Trp^320^ in various human cancer cell lines, such as MDA-MB-231, PC9, H3122, and HT1080.

### DPY19 family proteins are not *C*-mannosyltransferases of AXL

**Fig. 4:**
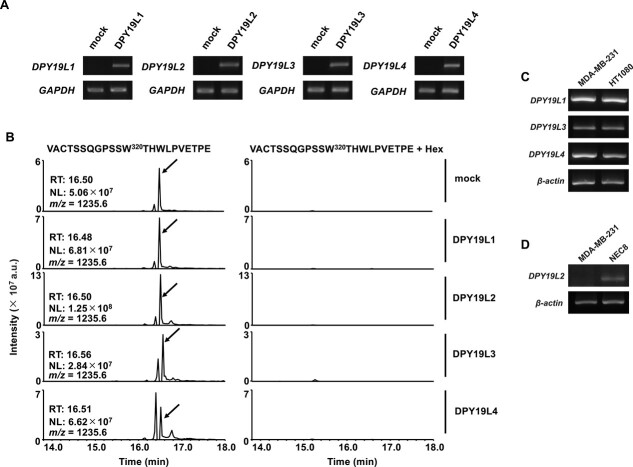
DPY19 family proteins are not *C*-mannosyltransferases of AXL. (A) Establishment of AXL-ECD and DPY19 family-co-expressing S2 cells. S2 cells were transfected with pMT-PURO-AXL-ECD and pIZ-DPY19L1, L2, L3, or L4. mRNA expression levels of DPY19 family were measured by RT-PCR. Mock S2 cells were co-transfected with pMT-PURO-AXL-ECD and pIZ/V5-his empty vector. (B) *C*-mannosylation of AXL is not catalyzed by DPY19 family proteins. Recombinant AXL-ECD was purified from conditioned media of DPY19 family-expressing S2 cells. These samples were analyzed by LC–MS/MS. Only unmodified ^309^VACTSSQGPSSWTHWLPVETPE^330^ peptides were detected in samples from mock and DPY19L1-, L2-, L3-, and L4-expressing cells as *m*/*z* = 1235.6. (C and D) mRNA expression level of DPY19 family in MDA-MB-231 cells. cDNA of HT1080 and NEC8 cells were used as a positive control of DPY19L1, L3, and L4 (HT1080, C), and DPY19L2 (NEC8, D).

The mammalian DPY19 family includes DPY19L1 to L4, of which DPY19L1 and L3 have *C*-mannosyltransferase activity in proteins with TSR domains. It is unclear whether DPY19L1 and L3 function as *C*-mannosyltransferases in proteins without TSR domains or whether DPY19L2 and L4 have enzymatic activity. To examine whether AXL is *C*-mannosylated by DPY19 family proteins, we used *Drosophila* S2 cells that lack *C*-mannosyltransferase activity. First, we established AXL-ECD-overexpressing S2 cells. Next, these cells were transiently transfected with human *DPY19L1-L4* genes, respectively (Fig. 4A). All recombinant AXL-ECD samples were purified and subjected to LC–MS/MS analysis (Fig. 4B). The LC–MS chromatograms showed that only unmodified ^309^VACTSSQGPSSWTHWLPVETPE^330^ peptides were detected from all samples. RT-PCR showed that DPY19L1, L3, and L4 were expressed in MDA-MB-231 cells (Fig. 4C), but not DPY19L2 (Fig. 4D). These results suggested that no DPY19 family protein was the *C*-mannosyltransferase of AXL.

### Depletion of *C*-mannosylation in AXL inhibits VM formation of MDA-MB-231 cells independently of activation of downstream signaling

**Fig. 5:**
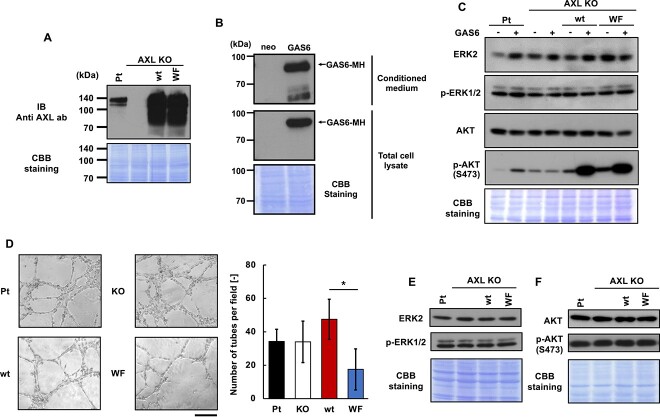
Effect of *C*-mannosylation of AXL in MDA-MB-231 cells. (A) Establishment of AXL knockout (AXL KO), re-expressing wild-type AXL (wt), and W320F mutant (WF) MDA-MB-231 cells. Parental (Pt), AXL KO, wt, and WF MDA-MB-231 cells were lysed, and each cell lysate was electrophoresed and immunoblotted with anti-AXL antibody. (B) Establishment of GAS6-overexpressing CHO-K1 cells. CHO-K1 cells transfected with pCI-neo empty vector (CHO-K1-neo) and GAS6-overexpressing CHO-K1 cells (CHO-K1-GAS6) were cultured in serum-free medium for 24 h. The cell lysates and conditioned media were electrophoresed and immunoblotted with anti-c-myc antibody. (C) Effect of *C*-mannosylation of AXL on its downstream signaling under GAS6 stimulation. Parental, AXL KO, wt, and WF MDA-MB-231 cells were cultured with serum-free DMEM for 24 h. These cells were treated with conditioned medium of CHO-K1-neo (GAS6 -) or CHO-K1-GAS6 (GAS6 +) for 15 min. Each cell lysate was electrophoresed and immunoblotted with anti-ERK2, anti-phospho-ERK1/2 (Thr202/Tyr204), anti-AKT, and anti-phospho-AKT (Ser473) antibodies. (D) VM formation assay of parental, AXL KO, wt, and WF MDA-MB-231 cells. These cells were seeded on Matrigel-coated well plates. Photographs were taken and the quantification of VM formation was performed at 5 h after seeding. Scale bar, 200 μm. Data shown are means ± SD. *P < 0.05. (E and F) Evaluation of phosphorylation levels of ERK (E) and AKT (F) of parental, AXL KO, wt, and WF MDA-MB-231 cells under normal culture conditions. Each cell lysate was electrophoresed and immunoblotted with anti-ERK2, anti-phospho-ERK1/2 (Thr202/Tyr204), anti-AKT, and anti-phospho-AKT (Ser473) antibodies.

To investigate the effect of *C*-mannosylation in AXL, we established AXL KO MDA-MB-231 cells and re-expressed wild-type AXL (wt) or a *C*-mannosylation-defective Trp320Phe mutant (WF) in AXL KO cells (Fig. 5A). To evaluate whether *C*-mannosylation of AXL affects the GAS6-induced signal activation, we established GAS6-overexpressing CHO-K1 cells (Fig. 5B). We treated parental, AXL KO, wt, and WF MDA-MB-231 cells with the conditioned medium of GAS6-overexpressing CHO-K1 cells and measured phosphorylation levels of ERK and AKT by GAS6 stimulation (Fig. 5C). Activation levels of ERK were not changed by GAS6 in all 4 cells analyzed. Activation of AKT by GAS6 was observed in AXL-positive cells, not in AXL KO cells. However, GAS6-induced phosphorylation of AKT was not changed by the presence or absence of *C*-mannosylation in AXL. This indicated that *C*-mannosylation of AXL does not affect the GAS6-induced signal activation.

Next, we performed VM formation assay using parental, AXL KO, wt, and WF MDA-MB-231 cells (Fig. 5D). In contrast to the results of the previous study, there was no difference in VM formation between parental and AXL KO cells. Interestingly, re-expression of AXL WF mutant in AXL KO cells induced the inhibition of VM formation. This result suggested that inhibition of *C*-mannosylation in AXL negatively regulates VM formation. To investigate whether the inhibition of VM formation caused by the depletion of *C*-mannosylation in AXL resulted from downstream signaling of AXL, the activation of downstream signaling was evaluated. Phosphorylation levels of ERK and AKT under normal culture conditions were assessed by western blot, but no difference in phosphorylation levels was observed between MDA-MB-231 cells with and without *C*-mannosylation of AXL (Fig. 5E and F). These data suggested that VM formation induced by *C*-mannosylation of AXL was not regulated by upregulation of its downstream signaling pathway.

## Discussion

Most of the *C*-mannosylation has been reported in proteins with TSR domains or belonging to the type 1 cytokine receptor family, and reports and functional analyses in proteins not belonging to these families are less advanced. In recent years, proteins that are *C*-mannosylated, even though they do not fit the consensus sequence, have been discovered, and conversely, cases in which *C*-mannosylation has not been discovered, even though they have the consensus sequence, have been reported (Osada et al. 2020; Hütte et al. 2022). Given this background, the discovery of *C*-mannosylation in domains other than those in which *C*-mannosylation frequently occurs could contribute to the advancement of *C*-mannosylation studies.

In this study, we were able to discover *C*-mannosylation in AXL protein, which does not have a TSR domain and is not a type 1 cytokine receptor. This is also the first discovery of *C*-mannosylation in RTKs. Furthermore, a *C*-mannosylation-defective mutant of AXL inhibited VM formation in MDA-MB-231 cells. It is hoped that the findings of this study will lead to further elucidation of the conditions and functions of *C*-mannosylation.

We attempted to verify whether the DPY19 family is the catalytic enzyme for *C*-mannosylation of AXL using *Drosophila* S2 cells, but none of the DPY19L1 to L4 proteins *C*-mannosylated AXL. In the mammalian DPY19 family, DPY19L1 and L3 have been reported to have *C*-mannosyltransferase activity. The *C*-mannosylated proteins known as substrates for DPY19L1 and L3 so far all belong to the TSR superfamily. Previous studies have also examined whether proteins that are not members of the TSR superfamily can be substrates of the DPY19 family, but all have been unsuccessful (Otani et al. 2018; Mizuta et al. 2023). Our results on AXL strongly suggest the existence of *C*-mannosyltransferases other than the DPY19 family.

Mass spectrometry results showed that *C*-mannosylation of AXL was not seen in all proteins. Not only is AXL not fully mannosylated, but many of the *C*-mannosylated proteins without TSR domains are not, either. The results of AXL and previous studies indicate that the inhibition of *C*-mannosylation by WF mutations affects protein function, suggesting that control of the amount of *C*-mannosylation may be controlling the function of the protein.

In vitro VM formation assay showed that depletion of *C*-mannosylation of AXL induced inhibition of VM formation in MDA-MB-231 cells. AXL was localized on the cell membrane in both wt and WF cells (Fig. S2), suggesting that AXL is rapidly transported to the cell membrane with or without *C*-mannosylation. Therefore, we concluded that localization is not the reason why *C*-mannosylation of AXL affects VM formation. Next, we predicted that the mechanism by which *C*-mannosylation of AXL affects VM formation is related to downstream signaling of AXL. However, it is suggested that *C*-mannosylation of AXL is not important for activation of major downstream signaling. These data indicate that the regulation of VM formation by *C*-mannosylation of AXL may be mediated through a pathway distinct from AXL downstream signaling. We attempted to detect the expression of EMT-related proteins, which are key factors in cell migration, invasion, and VM, for the MDA-MB-231 cells used in this study. However, expression levels of N-cadherin, E-cadherin and vimentin were not altered by KO of AXL, and wt or WF AXL re-expression (Fig. S3). These results suggest that the factor contributing to the difference in VM formation depending on whether AXL is *C*-mannosylated or not is an unknown mechanism that is neither dependent on downstream signaling of AXL nor on EMT.

Several of the proteins have been suggested to undergo conformational changes by *C*-mannosylation (Frank et al. 2020; Yoshimoto et al. 2023). Trp^320^ of AXL is in the fibronectin type-III domain, which is separated from the Ig-like domains that are important for its interaction with GAS6. This suggests that *C*-mannosylation does not affect GAS6-stimulated AXL activation. On the other hand, *C*-mannosylation of AXL affected VM formation in a downstream signaling-independent manner, suggesting that the conformational changes caused by AXL *C*-mannosylation loss affected its interactions with uncharacterized VM-related factors.

## Material and methods

### Cell lines

Human breast cancer MDA-MB-231 (gifted by Professor Masakazu Toi, Kyoto University Graduate School of Medicine, Kyoto, Japan), human fibrosarcoma HT1080 (Japanese Collection of Research Bioresources Cell Bank, Osaka, Japan), and human hepatoblastoma HepG2 (RIKEN BioResource Center, Tsukuba, Japan) were cultured in Dulbecco’s modified Eagle’s medium (DMEM), supplemented with 10% fetal bovine serum, 100 mg/L kanamycin, 100 units/mL penicillin G, 600 mg/L L-glutamine, and 2.25 g/L NaHCO_3_, at 37 °C with 5% CO_2_. Human NSCLC PC9 (American Type Culture Collection, Manassas, VA) and H3122 cells (gifted by Dr. JA Engelman, Massachusetts General Hospital Cancer Center, Boston, MS), and human embryonal carcinoma NEC8 cells (RIKEN BioResource Center) were cultured in Roswell Park Memorial Institute (RPMI) 1640 medium, supplemented with 10% fetal bovine serum, 100 mg/L kanamycin, 100 units/mL penicillin G, 300 mg/L L-glutamine, and 2.25 g/L NaHCO_3_, at 37 °C with 5% CO_2_. Human embryonic kidney HEK293T cells (RIKEN BioResource Center) were cultured in DMEM or RPMI 1640, depending on the application.

Chinese hamster ovary CHO-K1 cells (RIKEN BioResource Center) were cultured in Ham’s F-12 medium, supplemented with 10% fetal bovine serum, 100 mg/L kanamycin, and 100 units/mL penicillin G, at 37 °C with 5% CO_2_. *Drosophila melanogaster* embryo S2 cells (RIKEN BioResource Center) were cultured in Schneider’s *Drosophila* medium, supplemented with 10% heat-inactivated fetal bovine serum, 100 mg/L kanamycin, and 100 units/mL penicillin G, at 25 °C.

### Establishment of AXL-ECD-overexpressing cells

Human *AXL* gene was amplified from the pHAGE-AXL vector, which was purchased from Addgene (#116714). We amplified AXL-ECD using the following primers: 5’-TTTTGAATTCATGGCGTGGCGGTGCCCCAG-3′ (forward), 5’-AGATGAGTTTTTGTTCCCACCAGGGCCACGAGAAG-3′ (reverse). To introduce a myc-His_6_ tag into the C-terminus of AXL-ECD, we performed PCR with myc and His_6_ codon primers. The sequences of these tags were as follows: myc: 5’-GAACAAAAACTCATCTCAGAAGAGGATCTG-3′ and His_6_: 5’-CATCATCACCATCACCAT-3′. The amplified AXL-ECD gene was subcloned into the CSII-CMV-MCS-IRES2-Bsd vector (RIKEN BioResource Center). All plasmids constructed in this study have been analyzed by Macrogen Japan Corp. (Tokyo, Japan) to ensure that the DNA sequences are correct.

For lentivirus production, CSII-CMV-MCS-IRES2-Bsd-AXL-ECD plasmids were transfected into HEK293T cells using the Lentiviral High Titer Packaging Mix (Takara Bio Inc., Shiga, Japan). After 6 h of transfection, cells were washed, and fresh medium was added. After an additional 48 h of culture, the lentivirus-containing conditioned media was collected, and MDA-MB-231, HT1080, PC9, and H3122 cells were infected with the lentivirus-containing medium supplemented with 8 μg/mL polybrene. After infection, cells were selected with 10 μg/mL blasticidin S.

### Western blot

Western blot was performed using a slightly modified version of previously described methods (Simizu et al. 2004; Kawahara et al. 2018). Cells were cultured and lysed in a lysis buffer (50 mM Tris–HCl, pH 7.5, 150 mM NaCl, 0.1% (w/v) SDS, 1% (v/v) Triton X-100, 1% (w/v) sodium deoxycholate, and 1 mM phenylmethylsulfonyl fluoride [to detect protein phosphorylation only; phosphatase inhibitor cocktail PhosSTOP [Roche, Basel, Switzerland] was then added]) at 4 °C with sonication. The lysates were centrifuged at 15,300 × *g* for 10 min at 4 °C, and the amount of protein in each cell lysate was measured by Coomassie Brilliant Blue (CBB) G-250 (Bio-Rad Laboratories, Hercules, CA, USA). Then, the sample buffer (350 mM Tris–HCl pH 6.8, 30% (v/v) glycerol, 0.012% (w/v) bromophenol blue, 6% (w/v) SDS, and 30% (v/v) 2-mercaptoethanol) was added to each cell lysate, which was subsequently boiled at 98 °C for 3 min and electrophoresed on SDS-polyacrylamide gels. The proteins were transferred to a polyvinylidene difluoride membrane and immunoblotted with anti-c-myc (#9E10, Developmental Studies Hybridoma Bank, Iowa City, IA, USA), anti-AXL (#4566, Cell Signaling Technology, Danvers, MA, USA), anti-AKT (#9272, Cell Signaling Technology), anti-phospho-AKT (Ser473) (#4060, Cell Signaling Technology), anti-ERK2 (#sc1647, Santa Cruz Biotechnology, Dallas, TX, USA), anti-phospho-ERK1/2 (Thr202/Tyr204) (#9101, Cell Signaling Technology), anti-N-cadherin (#sc59987, Santa Cruz Biotechnology), anti-E-cadherin (#sc21791, Santa Cruz Biotechnology), or anti-vimentin (sc6260, Santa Cruz Biotechnology). Signals were detected by enhanced chemiluminescence using Western Lightning Plus-ECL (PerkinElmer, Inc., Waltham, MA, USA) or Immobilon Western Chemiluminescent HRP substrate (Merck KGaA, Darmstadt, Germany).

To detect the secretion of His_6_-tagged proteins, cells were washed with PBS and cultured in serum-free medium for 24 h. Conditioned media (CM) and cell lysates were collected. The CM was concentrated by Ni-NTA agarose (Roche Diagnostics, Mannheim, Germany) for 2 h at 4 °C, and the Ni-NTA agarose was collected and washed with buffer A (300 mM NaCl, 2.7 mM KCl, 10 mM Na_2_HPO_4_, 1.8 mM KH_2_PO_4_, and 5 mM imidazole). The imidazole concentration of buffer A was changed to 250 mM for elution of AXL-ECD from the Ni-NTA agarose. The proteins were electrophoresed on SDS-polyacrylamide gels and detected by western blot.

### Purification of recombinant AXL-ECD for LC–MS

AXL-ECD-overexpressing cells were cultured in serum-free medium for 24 h. The conditioned medium was collected and concentrated using Amicon Ultra-15 mL filters (Merck KGaA). The samples were further purified with Ni-NTA agarose. The eluates were electrophoresed on SDS-polyacrylamide gels, and protein bands were visualized by CBB staining.

### LC–MS/MS

Mass spectrometry was performed according to our previous study (Niwa et al. 2016). Briefly, protein bands corresponding to recombinant AXL-ECD were reduced with 50 mM DTT and then alkylated with 100 mM acrylamide. The bands were digested with 20 ng trypsin (TPCK treatment, Worthington Biochemical) and 20 ng endoprotease Glu-C (Roche) in 10 mM Tris–HCl (pH 8.0), 0.05% n-dodecyl-β-D-maltoside at 37 °C for 12 h. The peptides were subjected to liquid chromatography–tandem mass spectrometry (LC–MS/MS), consisting of an Easy nLC 1,000 and Q Exactive (Thermo Fisher Scientific Inc.), or an Easy-nLC 1200 and Q Exactive H-FX (Thermo Fisher Scientific Inc.). Peptides were separated using 0.1% formic acid as LC solvent in solvent A and 100% acetonitrile, 0.1% formic acid in solvent B, with linear gradients of 1%–50% or 1%–60% solvent B for 0–20 min. Each mass spectrometer was operated in positive-ion mode, and the spectra were acquired using a data-dependent top-10 method. The acquired spectra were analyzed against the in-house database with Mascot v.2.8 software (Matrix Science) and Proteome Discoverer 3.0 (Thermo Fisher Scientific Inc.). Mascot searches were performed using the following parameters: type of search- MS/MS ion search; enzyme- trypsin and V8-DE; fixed modification- none; variable modifications- acetyl (protein N-term), Gln → pyro-Glu (N-term Q), oxidation (M), propionamide (C), and Hex (W); mass values- monoisotopic; peptide mass tolerance- ±15 ppm; fragment mass tolerance- ±30 mmu; max missed cleavages- 4; and instrument type- ESI-TRAP. The MS chromatograms corresponding to unmodified and mono-hexosylated peptides were plotted using Qual browser 4.1.31.9 (Thermo Fisher Scientific Inc.).

### Protein expression in S2 cells

*AXL*-ECD gene was amplified from the CSII-CMV-MCS-IRES2-Bsd-AXL-ECD vector using the following primers: 5’-TTTTGATCTGCCCCCAGGGGCACGCAC-3′ (forward), 5’-TTTTACGCGTCAGATCCTCTTCTGAGATGAG-3′ (reverse). The amplified *AXL*-ECD gene was subcloned into the pMT-PURO vector (RIKEN BioResource Center). S2 cells were transfected with pMT-PURO-AXL-ECD using *Trans*IT-Insect Transfection Reagent (Mirus Bio LLC, Madison, WI, USA), followed by selection with 10 μg/mL puromycin. These cells were seeded and transiently transfected with pIZ-DPY19L1, L2, L3, and L4 (Niwa et al. 2016), respectively. After 6 h, the cells were washed and cultured in serum-free medium with 500 μM CuSO_4_ to induce AXL-ECD expression. After an additional 66 h of induction, the conditioned medium was collected and purified with Ni-NTA agarose. The samples were electrophoresed on SDS-polyacrylamide gels, and protein bands were visualized by CBB staining.

### RT-PCR

Cells were lysed, and isolated total RNAs were used for the reverse-transcription reaction using the High-Capacity cDNA Reverse Transcription Kit (Thermo Fisher Scientific Inc., Waltham, MA). The resulting cDNA was used for PCR. The primers to detect the expression of the human *DPY19* family, human *β-actin*, and fly *GAPDH* were previously designed in our studies (Niwa et al. 2016).

### Establishment of AXL knockout cells

AXL KO cells were established using the CRISPR/Cas9 system. The oligos to generate single-guide RNA (sgRNA) were inserted into the pSpCas9(BB)-2A-Puro (PX459) V2.0 plasmid (#62987, Addgene). The primers to construct AXL-targeting sgRNA were as follows: 5’-CACCGGGCTGTGCTGTCAGACGAT-3′ (forward), 5’-AAACATCGTCTGACAGCACAGCCC-3′ (reverse). The pair of primers was annealed and subcloned into the plasmid. The obtained plasmids were transfected into MDA-MB-231 or PC9 cells using Lipofectamine 3000™ (Thermo Fisher Scientific Inc.), and then, the cells were selected with 2 μg/mL puromycin. KO of AXL was confirmed by western blot.

### Establishment of Cas9-resistant AXL-overexpressing cells

Human *AXL* full length gene was amplified from the pHAGE-AXL vector. The sequences of the primers to amplify full-length AXL were as follows: 5’-TTTTGAATTCATGGCGTGGCGGTGCCCCAG-3′ (forward), 5′- TTTTGCGGCCGCTCAGGCACCATCCTC-3′ (reverse). To prevent Cas9 recognition and deletion of exogenously introduced *AXL* gene, we constructed Cas9-resistant *AXL* gene by inverse PCR with the following primers: 5’-GGCAGTCCTCTCTGATGACGGGATGGGCATCCAG-3′ (forward), 5’-TGCAGGGTGCAGTGGGTCAGGGGGTAGATG-3′ (reverse). We substituted Trp^320^ residues in AXL with Phe residues by inverse PCR. The sequences of primers for mutagenesis were as follows: 5’-TTCACCCACTGGCTTCCTGTGGAGAC-3′ (forward), 5’-GGATGAGGGGCCCTGGCTGCTGGTGCATG-3′ (reverse). The Cas9-resistant *AXL* gene was subcloned into the CSII-CMV-MCS-IRES2-Bsd vector. The methods of lentivirus construction, infection, and selection were the same as that of AXL-ECD expression.

### VM formation assay

The in vitro VM formation assay was conducted as described in our previous study (Kawahara et al. 2018; Shimizu et al. 2021; Kamo et al. 2022; Nakajima et al. 2023). MDA-MB-231 cells were seeded at 1.6 × 10^4^ cells/well into a 96-well plate, which was precoated with 40 μL/well Matrigel (Corning Inc., Corning, NY, USA), and cultured at 37 °C with 5% CO_2_. In each well, photographs of 4 independent, randomly selected fields were taken using phase-contrast microscopy (Leica DMi1, Leica, Wetzlar, Germany), and the number of tubes was counted.

### Evaluation of AXL localization by immunofluorescence

We inserted a myc-tag just after the AXL signal peptide sequence in the Cas9-resistant AXL (wt/WF) overexpression plasmids by PCR. The sequences of the primers to insert myc-tag were as follows: 5’-ATCTCAGAAGAGGATCTGGCCCCCAGGGGCAC-3′ (forward), 5’-GAGTTTTTGTTCCATGCACGCCCAGCCGCAC -3′ (reverse). The Cas9-resistant myc-*AXL* gene was subcloned into the CSII-CMV-MCS-IRES2-Bsd vector. The methods of lentivirus construction, infection, and selection were the same as that of other cell lines in this study.

AXL-KO/myc-AXL (wt/WF) overexpressing MDA-MB-231 cells were cultured on coverslips. After 24 h, the cells were washed with PBS, fixed with 4% paraformaldehyde for 10 min and permeabilized with 0.1% (v/v) Triton X-100 for 10 min. After blocking with 3% bovine serum albumin, the cells were incubated with anti-c-myc for 1 h. Alexa Fluor 488-conjugated anti-mouse IgG (Thermo Fisher Scientific, Inc.) was used as the secondary antibody. To detect the Golgi apparatus and ER, the cells were incubated with anti-GRASP65 (#sc30093, Santa Cruz Biotechnology, Inc.) and anti-calnexin (#2679, Cell Signaling Technology, Inc.) as primary antibodies, respectively, and then incubated with Alexa Fluor 568-conjugated anti-rabbit IgG (Thermo Fisher Scientific, Inc.) as the secondary antibody. When evaluating the localization of AXL on the cell membrane, the cells were stained with c-myc antibody without permeabilization by Triton X-100.

### GAS6 stimulation using GAS6-overexpressing CHO-K1 cells

Human *GAS6* gene was amplified from the pENTR-TOPO-GAS6 vector (RIKEN BioResource Center) using the primers as follows: 5’-TTTTGAATTCATGGCCCCTTCGCTCTCGCC-3’ (forward), 5’- GATGAGTTTTTGTTCGGCTGCGGCGGGCTC-3’ (reverse). To introduce a myc-His_6_ tag into the C-terminus of GAS6, we performed PCR with myc and His_6_ codon primers. The amplified *GAS6* gene was subcloned into the pCI-neo vector (Promega Corporation, Madison, WI, USA). pCI-neo-GAS6 plasmids were transfected into CHO-K1 cells using Lipofectamine 3000™, and then, the cells were selected with 400 μg/mL G418.

GAS6-overexpressing CHO-K1 cells were cultured with serum-free medium for 24 h. MDA-MB-231 cells cultured in serum-free DMEM for 24 h were treated with the conditioned medium of GAS6-overexpressing CHO-K1 cells for 15 min, and then, cell lysates were collected. As a negative control, the conditioned medium from CHO-K1 cells transfected with pCI-neo empty vector (CHO-K1-neo) (Okamoto et al. 2017) was used.

### Statistical analysis

All data except LC–MS/MS are representative of three separate experiments. Datasets were analyzed using one-way ANOVA with Tukey’s test using SPSS (version 29; IBM, Armonk, NY). The results were expressed as mean ± SD. *P* < 0.05 was considered to indicate a statistically significant difference.

## Supplementary Material

Supplementary_data_Final_cwae096

## Data Availability

The data supporting the findings of this study are available from the corresponding author upon reasonable request.

## Supplementary material

LC–MS analysis of recombinant AXL-ECD in PC9, H3122, and HT1080 cells, *C*-mannosylation affects neither subcellular localization nor translocation to the plasma membrane of AXL protein, Expression of AXL and the presence or absence of *C*-mannosylation do not affect the expression of EMT marker proteins in MDA-MB-231 cells.
