# Supplementary material for: Identification of C-mannosylation in a receptor tyrosine kinase AXL
Source: Glycobiology. 2024 Dec 3;34(11):cwae096. doi: 10.1093/glycob/cwae096 (PMC11632359; doi:10.1093/glycob/cwae096)
Supplement: Supplementary_data_Final_cwae096 [file supplementary_data_final_cwae096.pdf]

Identification of *C*-mannosylation in a receptor tyrosine kinase AXL

Kento Mori[1], Takehiro Suzuki[2], Urara Waki[1], Soichiro Hayashi[1], Shigehito Kadono[1], Ryota Kawahara[1], Minae Takeuchi[1], Hayato Mizuta[1,3], Naoshi Dohmae[2], Ryohei Katayama[3], Siro Simizu[1]

[1] Department of Applied Chemistry, Faculty of Science and Technology, Keio University, Yokohama, Japan
[2] Biomolecular Characterization Unit, RIKEN Center for Sustainable Resource Science, Saitama, Japan
[3] Division of Experimental Chemotherapy, Cancer Chemotherapy Center, Japanese Foundation for Cancer Research, Tokyo, Japan

Supporting Data

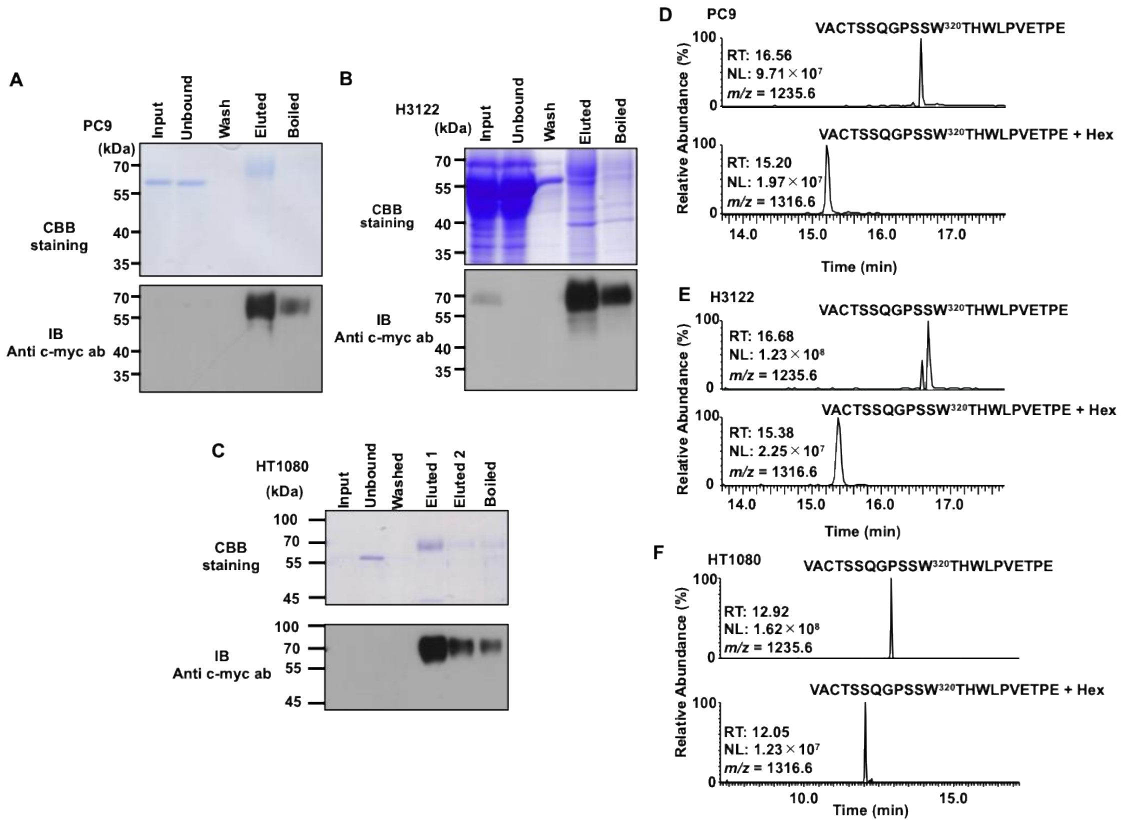

Fig. S1. LC-MS analysis of recombinant AXL-ECD in PC9, H3122, and HT1080 cells. (A-C) Purification of recombinant AXL-ECD from AXL-ECD-overexpressing PC9 (A), H3122 (B), and HT1080 (C) cells. Cells were cultured in serum-free DMEM for 24 h, and conditioned medium was collected. The conditioned medium was concentrated by ultrafiltration and purified with Ni-NTA agarose. The samples were electrophoresed, and the bands were visualized by CBB staining. (D-F) LC-MS chromatograms of the unmodified and hexosylated ^{309}VACTSSQGPSSWTHWLPVETPE^{330} peptides derived from AXL-ECD-overexpressing PC9 (D), H3122 (E), and HT1080 (F) cells. The unmodified and hexosylated ^{309}VACTSSQGPSSWTHWLPVETPE^{330} peptides were detected as *m*/*z* = 1235.6 and 1316.6, respectively.

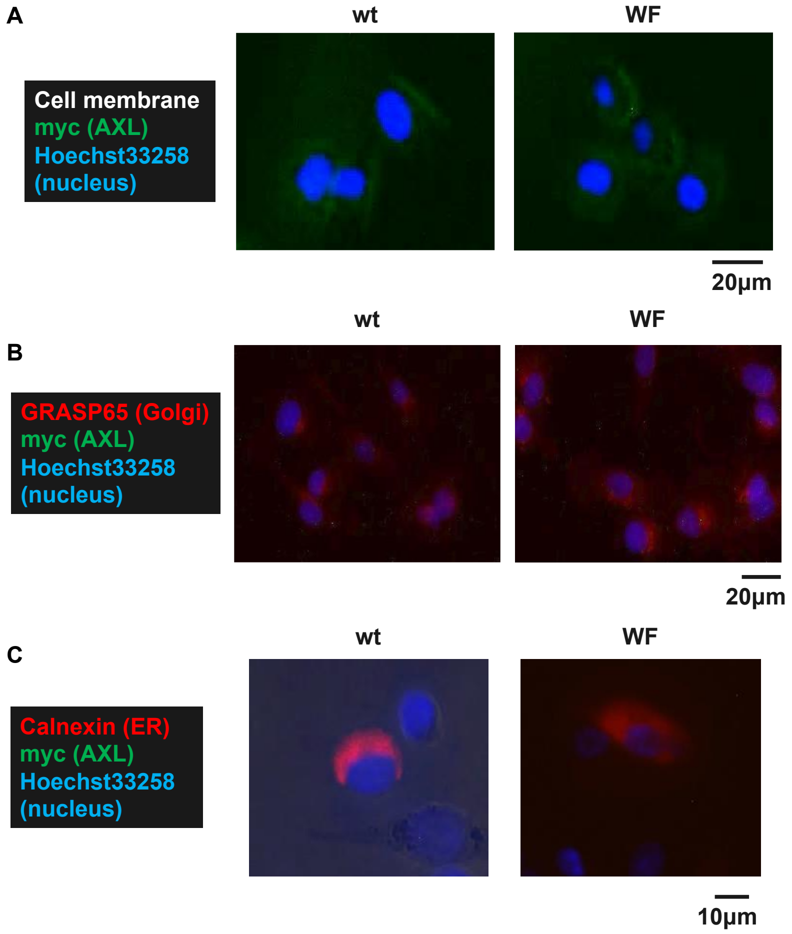

Fig. S2. *C*-mannosylation affects neither subcellular localization nor translocation to the plasma membrane of AXL protein.

(A-C) Immunofluorescence of myc-AXL overexpressing MDA-MB-231 cells. Myc-tagged AXL (wt or WF) was overexpressed in AXL-KO MDA-MB-231 cells. To label myc-AXL on the cell membrane, cells were stained with anti-myc antibody without membrane permeabilization (A). To evaluate the subcellular localization of myc-AXL, cells were permeabilized and stained with anti-myc and anti-GRASP65 (B) or anti-calnexin (c) antibodies.

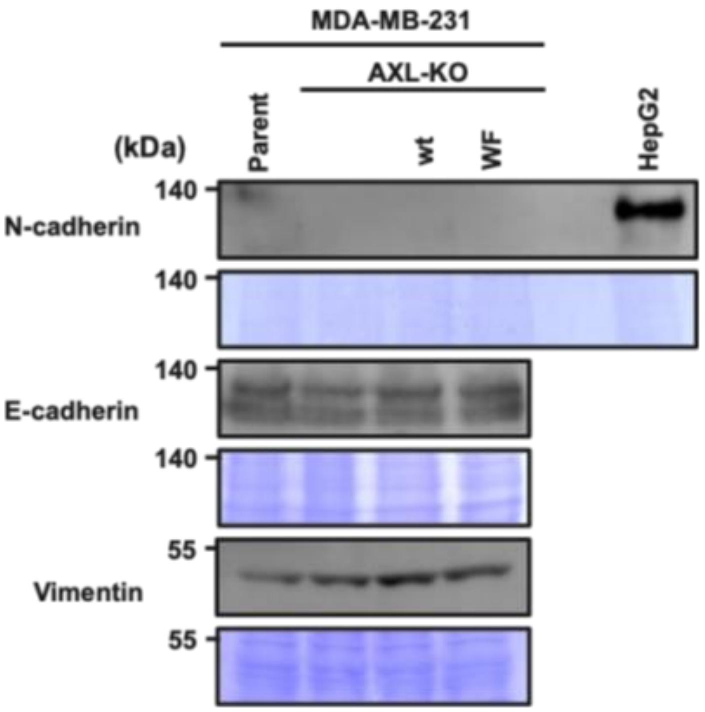

Fig. S3. Expression of AXL and the presence or absence of *C*-mannosylation do not affect the expression of EMT marker proteins in MDA-MB-231 cells.

Evaluation of expression levels of N-cadherin, E-cadherin, and vimentin of parental, AXL KO, wt, and WF MDA-MB-231 cells under normal culture conditions. Each cell lysate was electrophoresed and immunoblotted with anti-N-cadherin, anti-E-cadherin, and anti-vimentin antibodies. The cell lysate of HepG2 cells was used as the positive control of N-cadherin expression.
